# Tamoxifen attenuates postsurgical hindlimb swelling and soft-tissue fibrosis in a rat model of secondary lymphatic injury

**DOI:** 10.1007/s00210-026-05377-8

**Published:** 2026-05-09

**Authors:** Ertugrul Kargi, Turgay Simsek, Nuh Zafer Canturk, Mustafa Dulger, Hakan Demir, Mustafa Cekmen, Cengiz Ercin

**Affiliations:** 1 Department of General Surgery, Kocaeli University School of Medicine, Kocaeli, Turkey; 2 Experimental Medicine and Research Laboratory, Kocaeli University School of Medicine, Kocaeli, Turkey; 3 Department of Nuclear Medicine, Kocaeli University School of Medicine, Kocaeli, Turkey; 4 Department of Biochemistry, Kocaeli University School of Medicine, Kocaeli, Turkey; 5 Department of Pathology, Kocaeli University School of Medicine, Kocaeli, Turkey

**Keywords:** Antifibrotic effect, Axillary dissection, Breast cancer, Lymphedema, Rat, Tamoxifen

## Abstract

Secondary lymphedema is a common complication after lymph node dissection and is characterized by chronic swelling and progressive soft-tissue fibrosis. Tamoxifen has been reported to exert antifibrotic and antioxidant effects. We investigated whether tamoxifen could attenuate postsurgical limb swelling and fibrosis in a rat model of secondary lymphatic injury and explored potential oxidative mechanisms. Forty Wistar–Albino rats were randomized into four groups (*n* = 10 each). A standardized right hindlimb lymphadenectomy was performed in all groups. Tamoxifen (2.5 mg/kg/day, oral) was administered with different schedules: 7 days preoperative + 7 days postoperative (Group 2), 14 days postoperative starting immediately (Group 3), and 14 days postoperative starting on day 14 (Group 4). Outcomes at day 30 included limb volume change (water volumetry), lymphoscintigraphy (Tc-99 m nanocolloid), histopathological fibrosis score, tissue hydroxyproline, oxidative stress markers, malondialdehyde (MDA) and glutathione (GSH), and selected cytokines. Lymphoscintigraphic parameters did not differ significantly between groups. However, postoperative volumetric increase of the operated limb was significantly greater in untreated controls than in Groups 2 and 3 (*p* < 0.05). Histopathologic fibrosis scores were significantly lower in Groups 2 and 3 compared to the controls, supported by lower hydroxyproline levels. Tissue MDA levels were higher and GSH levels lower in controls compared with treated groups., suggesting attenuation of oxidative stress by tamoxifen. In this rat model, tamoxifen reduced postsurgical limb swelling and soft-tissue fibrosis, accompanied by lower oxidative stress. However, lymphoscintigraphy did not demonstrate significant between-group differences and a sham-operated control group was not included. Thus, our findings support an antifibrotic/antioxidant effect of tamoxifen on postoperative tissue changes rather than lymphedema prevention.

## Introduction

Axillary dissection is commonly performed in women undergoing surgery for breast cancer. Although it does not predict the survival of these patients, axillary lymph node status is still a valuable prognostic indicator and an effective measure for the control of regional disease (Abu-Rustum et al. 2006). However, one of the most significant complications after axillary dissection is lymphedema of the ipsilateral upper extremity, which may cause functional loss, physical discomfort, and cosmetic disfigurement (Anuszkiewicz et al. 2023).

Despite the prevalence and morbidity of lymphedema, there are no proven preventative measures, with most currently available treatment options being supportive and noncurative. Although surgical approaches have been described, they have not gained widespread acceptance (Avraham et al. 2010). Impaired lymphatic regeneration and lymphatic function are a consequence of impaired lymphatic endothelial cell proliferation, an abnormal lymphatic microarchitecture, and lymphatic fibrosis, and these lead to fibrosis of the involved soft tissues. Inhibition of fibrosis using a simple topical dressing can markedly accelerate lymphatic repair and promote regeneration of the normal capillary lymphatics (Baik et al. 2022). Tamoxifen is a selective estrogen receptor modulator administered to breast cancer patients following radiotherapy and chemotherapy. It is also an antifibrotic agent and has been used in the treatment of retroperitoneal fibrosis and Riedel’s thyroiditis (Bocale et al. 2011; Bowman and Rockson 2024). The aim of the present study was to evaluate whether tamoxifen attenuated postoperative limb swelling and soft-tissue fibrosis following standardized hindlimb lymphatic injury in rats and to explore potential pathogenetic mechanisms through assessment of oxidative stress and inflammatory mediators.

## Methods

### Animals and surgical procedure

Forty Wistar–Albino rats, weighing 200–250 g, were selected as experimental subjects. These animals were handled in compliance with the protocol of the Experimental Medicine and Research Laboratory of Kocaeli University (DETAB), Turkey. This study was approved by the Ethics Committee for Animal Research of Kocaeli University, funded by Kocaeli University Research Fund with the number 2014/0012, and performed in accordance with internationally accepted standard guidelines for the care and use of laboratory animals. Rats were housed in stainless steel cages at a room temperature of 18–26 °C and a relative humidity of 40–60% and were provided with food and water ad libitum. The rats were randomly divided into four groups of 10. These were the control group (Group 1) and three experimental groups (Groups 2, 3, and 4). Groups 2, 3, and 4 received the same dose and duration of tamoxifen therapy, but it was administered at different time points to determine the optimal schedule for preventing or alleviating lymphedema. Tamoxifen was administered through an orogastric tube at a dose of 2.5 mg/kg/day. Group 2 received the drug for 7 days preoperatively and 7 days postoperatively. Group 3 received the drug for 14 days postoperatively, with treatment starting immediately following lymphadenectomy. Group 4 received the drug for 14 days postoperatively, from day 14 to day 30 of the experiment. The rationale for these treatment timing differences between the groups is as follows. Preoperative plus early postoperative administration (Group 2) represents a pharmacological preconditioning strategy designed to modulate the inflammatory cascade before lymphatic injury and during the immediate postlymphadenectomy phase. Immediate postoperative treatment (Group 3) specifically targets the peak inflammatory response and early profibrotic signaling, making it the most pathogenesis-aligned and mechanistically synchronized preventive intervention. In contrast, delayed treatment (Group 4) simulates modulation of established fibrosis and therefore represents a therapeutic model aimed at assessing the reversibility of chronic lymphedematous changes, rather than prevention.

**Fig. 1:**
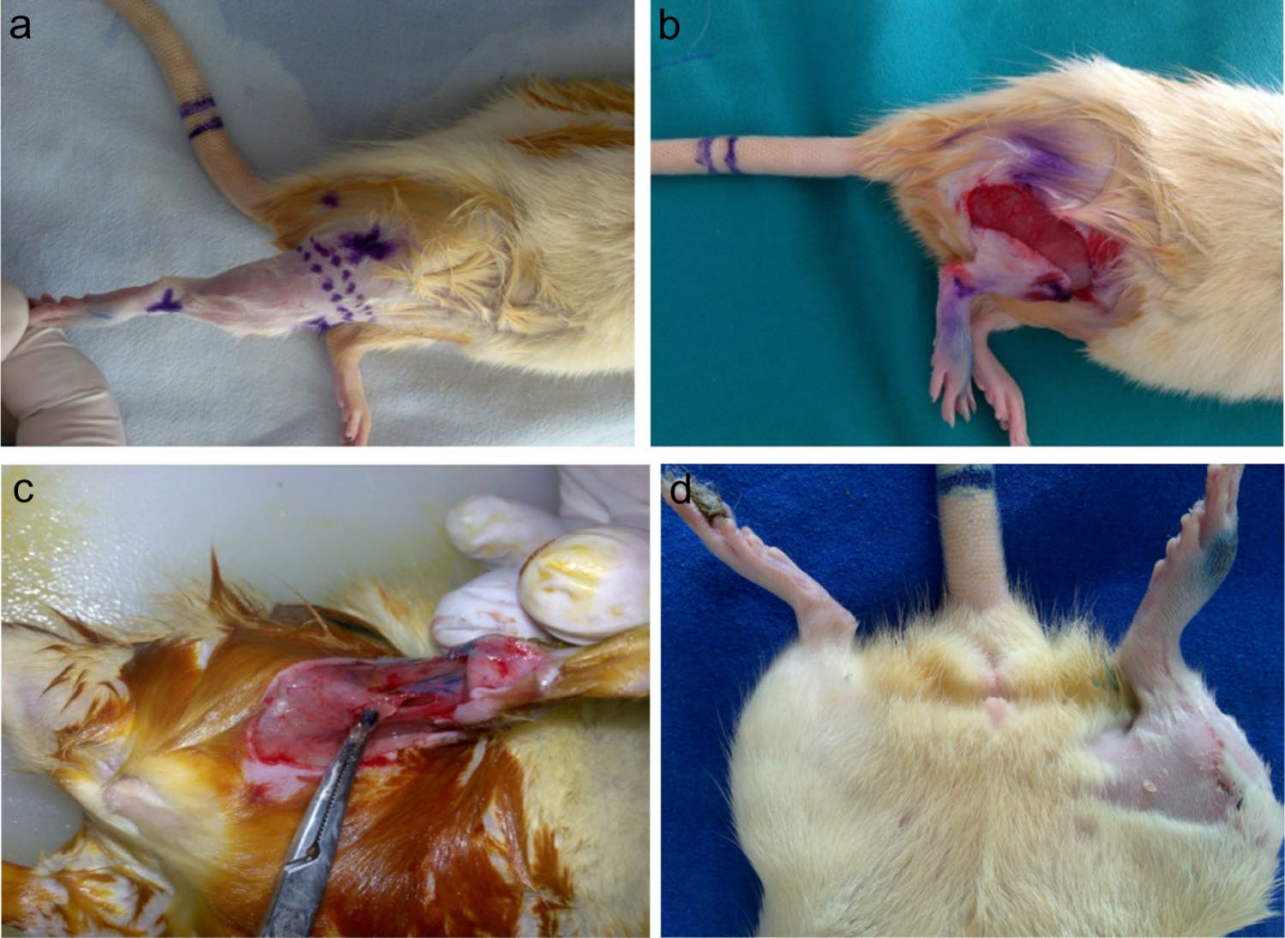
**a**, **b** Skin, subcutaneous tissue, and fascia from the proximal thigh were removed. **c** Lymph nodes and vessels, which had been stained with methylene blue, were removed segmentally and ligated. **d** A sample of developed lymphedema at the end of the 30th day

Animals were anesthetized with 50-mg ketamine hydrochloride per kg body weight (Ketalar, Pfizer) and 5-mg xylazine per kg (Rompun, Bayer), injected intraperitoneally. The right hind foot of each anesthetized rat was then subcutaneously injected with 0.5 ml of 0.5% methylene blue at neutral pH in saline solution, in order to visualize the peripheral lymph nodes. A 10-mm diameter portion of tissue was removed from the right proximal thigh. This excised tissue included skin, subcutaneous tissue, and underlying fascia. Lymph nodes and lymphatic vessels identified with methylene blue were removed segmentally and the proximal and distal borders were ligated. The wound was sutured with 2/0 silk, covered with an antibacterial gel, and bandaged. At the end of 30 postoperative days, lymphedema was evaluated by lymphoscintigraphy and volumetrically (Fig. 1a–d), as previously described (Brandt et al. 2014).

### Lymphoscintigraphy

Animals were anesthetized before lymphoscintigraphy using the same regimen described for the initial surgery. Three hundred microliters of 25 μCi Tc-99 m nanocolloid (Nanocis, Cisbio, France) was interdigitally injected into both hind limbs. The injected areas were massaged to distribute the Tc-99 m nanocolloid within the interdigital soft tissue. The rats were allowed to move freely in their cages for 5 min after recovery from the anesthesia. Dynamic and static images of both lower extremities were obtained with a single-head gamma camera (ADAC Argus Epic, ADAC Laboratories, Milpitas, California, USA) equipped with a low-energy, general-purpose, parallel hole collimator. All images were saved and evaluated quantitatively and qualitatively.

### Volume estimation

The volume of the right thigh of each rat was measured preoperatively and at the end of the experiment on postoperative day 30. Water volumetry was performed with a water-filled 50-ml graduated cylinder. The right thigh of each rat was immersed in a cylinder filled with 45 ml of water. The amount of water needed to bring the volume to 50 ml was used to calculate the thigh volume. The volume of each right thigh was measured three times, and the mean was taken.

### Histopathology

Tissue samples were fixed in 10% neutral formalin solution. After routine histopathological procedures, the specimens were embedded in paraffin blocks and cut into 5-µm-thick sections. The tissue sections were stained with hematoxylin and eosin and Mason triple stains and then photographed. Fibrosis was detected using a scoring method that assesses fibrosis, inflammation, and vascular proliferation, rated on a modified semi-quantitative scale of 0–3. The amount of fibrosis was scored as follows: 0, no fibrosis; 1, minimal, loose fibrosis; 2, moderate fibrosis; and 3, florid dense fibrosis (Davis and Bertram 2025). A score of 2 or 3 was assumed to indicate positivity for fibrosis.

### Biochemical analysis

The rats were euthanized on day 30. Blood collected directly from the heart of each animal was allowed to clot for 1 h at room temperature and then centrifuged to obtain the serum, which was stored at − 80 °C for later analysis. Tissue samples were also frozen and stored at − 80 °C until biochemical evaluation. Glutathione (GSH) levels were assayed by means of Ellman’s reagent. Malondialdehyde (MDA) levels were measured by reacting with thiobarbituric acid (TBA) followed by high-performance liquid chromatography separation of the MDA-TBA conjugate, as previously described (Duhon et al. 2022; Ersoy et al. 2009). NO measurements were done with the Griess method, previously described in literature (Gashew et al. 2002). Tissue hydroxyproline levels (Biovison) as an indicator of the formation of connective tissue, interleukin (IL)−1 and tumor necrosis factor-α (TNF-α) (Invitrogen), and tissue interferon (IFN)-γ levels (Biosource) concentrations were measured with commercial kits at the Alisei Quality System of Kocaeli University Central Laboratory.

### Statistical analysis

Statistical analysis was performed using SPSS, version 17.0 (IBM Inc., Armonk, NY, USA). The results are expressed as the means ± standard deviation (SD). Student’s *t*-test was used for comparison of the data. When appropriate, analysis of variance and Tukey’s test for multiple comparisons were used to analyze the biochemical data. Edema percentages were compared using a *χ*^2^test. Differences were considered statistically significant at *p* < 0.05.

## Results

**Fig. 2:**
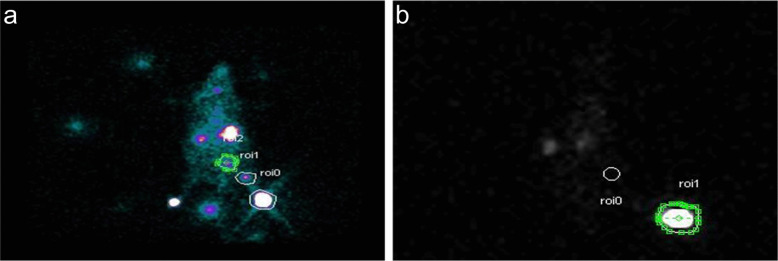
A sample of **a** positive lymphoscintigraphy and **b** negative lymphoscintigraphy

**Fig. 3:**
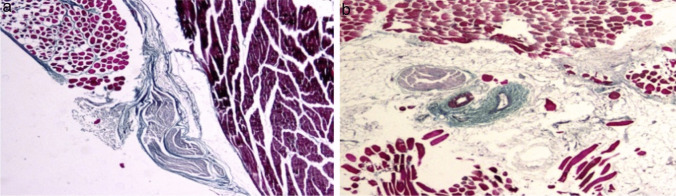
**a**, **b** Histopathological samples for lymphedema

At the end of the experiment, on day 30, the volumetric changes in the thighs of control rats in Group 1 were significantly higher (*p* < 0.05) than those of rats in experimental Groups 2 and 3 (Table 1). In addition, the thigh volume of rats in Group 4 was higher than that of rats in Group 2. Although visualization of iliac lymph nodes was used as an indicator of tracer transport through the lymphatic pathway, quantitative and qualitative lymphoscintigraphic assessments did not reveal significant between-group differences at day 30 (Fig. 2). Conventional histopathological evaluation showed that fibrosis scores were significantly lower in Groups 2 and 3 than in Group 1 (*p* = 0.005 and *p* = 0.033, respectively) (Fig. 3). Tissue hydroxyproline levels were lower in Group 2 than in the control group (*p* < 0.05) (Table 1), thus providing biochemical evidence to support the histopathological results.

**Table 1:** Lymphedema in tamoxifen-treated (Groups 2–4) and untreated control (Group 1) rats; *n* = 10 rats/group

	Scintigraphy^a^	Volumetric changes	Fibrosis^b^	Hydroxyproline
	+/−		+/−	
Group 1	6/4	2.70 ± 0.75	10/0	0.84 ± 0.15
Group 2	7/3	1.10 ± 0.61	4/6	0.68 ± 0.23
Group 3	4/5	1.72 ± 0.44	5/4	1.10 ± 0.40
Group 4	4/6	2.25 ± 0.68	7/3	0.92 ± 0.22

Hydroxyproline: control versus Group 2, *p* < 0.05; Group 2 versus Group 3, *p* < 0.01

Volumetric change: control versus Group 2, *p* < 0.001; control versus Group 3, *p* = 0.01; Group 2 versus Group 4, *p* = 0.002

^a^Number of rats positive or negative for scintigraphy determined by transport capacity of the injured lymph vessels

^b^Number of rats positive or negative for fibrosis, determined by the amount of fibrosis, was scored as follows: 0, no fibrosis; 1, minimal, loose fibrosis; 2, moderate fibrosis; and 3, florid dense fibrosis. A score of 2 or 3 indicated positivity for fibrosis. Fibrosis: control versus Group 2, *p* = 0.005; control versus Group 3, *p* = 0.033

Tissue nitric oxide (NO) did not significantly differ among the four groups, nor were there significant differences in blood NO and GSH levels. Tissue GSH levels were significantly higher in Groups 2 and 3 (*p* = 0.043 and *p* = 0.001, respectively) than in Group 1 and significantly lower (*p* < 0.005) in Group 4 than in Group 2 (Table 2). Tissue MDA levels were significantly lower in Groups 2, 3, and 4 compared to Group 1 (*p* = 0.001) and significantly higher in Group 4 than in Group 2 (*p* < 0.001). Similarly, serum MDA levels were significantly lower in Groups 2 and 3 than in Group 1 and significantly higher in Group 4 than in Groups 2 and 3 (Group 1 vs. Group 2, *p* < 0.001; Group 1 vs. Group 3, *p* < 0.001; Group 2 vs. Group 4, *p* < 0.001)_._ There was no significant difference in tissue cytokine levels, except for IL-1. IL-1 levels were higher in Group 2 than in Group 3 and significantly lower in Group 2 than in Group 1 (Table 3). Serum TNF levels were higher in Group 4 than in Groups 2 and 3 (*p* > 0.05) (Table 4).

**Table 2:** Tissue levels of nitric oxide (NO), malondialdehyde (MDA), and reduced glutathione (GSH) in tamoxifen-treated (Groups 2–4) and untreated control (Group 1) rats; *n* = 10 rats/group

	NO (nmol/g)	MDA (nmol/g)	GSH (nmol/g)
Group 1	98.43 ± 14.00	68.29 ± 18.2	3.52 ± 1.47
Group 2	84.55 ± 27.48	33.15 ± 11.55	6.46 ± 1.65
Group 3	107.23 ± 22.41	31.98 ± 13.77	5.46 ±.53
Group 4	86.67 ± 19.71	37.13 ± 9.66	4.12 ± 1.10

MDA: control versus Group 2, *p* < 0.001; control versus Group 3, *p* < 0.001; Group 2 versus Group 4, *p* < 0.001. GSH: control versus Group 3, *p* < 0.043; control versus Group 2, *p* < 0.001; Group 2 versus Group 4, *p* < 0.005

**Table 3:** Tissue cytokine levels in tamoxifen-treated (Groups 2–4) and untreated control (Group 1) rats; *n* = 10 rats/group

	IFN (IU/ml)	IL-1 (picogram/ml)	TNF (picogram/ml)
Group 1	1.70 ± 0.83	152.20 ± 48.38	99.74 ± 38.29
Group 2	1.015 ± 0.53	109.12 ± 27.03	85.24 ± 15.24
Group 3	1.49 ± 0.58	146.54 ± 47.29	116.76 ± 42.72
Group 4	1.52 ± 0.65	167.46 ± 38.46	98.09 ± 39.68

IL-1: Group 2 versus Group 4, *p* < 0.001

**Table 4:** Blood nitric oxide (NO), malondialdehyde (MDA), reduced glutathione (GSH), and tumor necrosis factor-α (TNF-α) levels in tamoxifen-treated (Groups 2–4) and untreated control (Group 1) rats; *n* = 10 rats/group

	NO (nmol/ml plasma)	MDA (nmol/ml plasma)	GSH (nmol/ml plasma)	TNF (picogram/ml plasma)
Group 1	36.52 ± 14.66	6.50 ± 1.44	1.16 ± 0.44	47.46 ± 18.68
Group 2	31.38 ± 9.88	3.90 ± 1.13	0.97 ± 0.28	41.18 ± 15.76
Group 3	36.62 ± 14.10	3.86 ± 1.44	0.90 ± 0.42	38.77 ± 7.72
Group 4	30.24 ± 10.07	6.96 ± 2.14	1.30 ± 0.46	59.33 ± 13.03

MDA: control versus Group 2, *p* < 0.004; control versus Group 3, *p* < 0.005; Group 3 versus Group 4, *p* < 0.001; Group 2 versus Group 4, *p* < 0.001. TNF: Group 2 versus Group 4, *p* < 0.047; Group 3 versus Group 4, *p* < 0.02

## Discussion

Secondary lymphedema is a debilitating complication after lymph node dissection. In an experimental context, postsurgical limb volume changes and progressive soft-tissue fibrosis are considered key features of lymphatic injury–related tissue remodeling. In the present study, lymphoscintigraphic assessments at day 30 did not demonstrate significant differences between groups. Therefore, our findings concerning the effect of tamoxifen treatment should be interpreted as attenuation of postoperative limb swelling and fibrosis rather than definitive prevention of lymphedema. Chronic lymphedema is a devastating disorder that occurs in 13–50% of patients undergoing lymph node dissection for breast cancer. It has a highly negative impact on the quality of life by impairing both function and appearance of the involved upper extremity(Gilkeson and Allen 1996). There is no commonly accepted preventive and therapeutic strategy (Avraham et al. 2010), although potential, preventative solutions have been described (Granger et al. 1996; Guo et al. 2014). Lymphedema develops because of the limited transport capacity of the injured lymph vessels, which leads to an increase in lymphatic volume and pressure and in the osmotic pressure of the interstitial space. The most important pathological changes in the lymphatic system and interstitial tissues of patients with lymphedema are dilatation of lymph vessels, functional and morphological injury of endothelial cells, and fibrosis and sclerosis of regional lymphatic networks (Hagendoorn et al. 2005; Huang 2014). Clinically, an association between lymphedema and fibrosis has been demonstrated (Itai et al. 2024; Jung et al. 2014; Kalinin 2021; Kareh and Xu 2020; Kataru et al. 2019). In the present study, experimental lymphedema was associated with fibrosis and increased levels of hydroxyproline, the latter being indicative of the formation of connective tissue.

The risk of lymphedema is higher in the presence of patient- and treatment-related factors that promote fibrosis, such as extensive surgical resection, infection, and radiation therapy. Several potential mechanisms may be responsible for the delayed lymphatic regeneration that occurs in the setting of soft-tissue fibrosis (Kootstra et al. n.d.; Kuhn et al. 2002; Kum-Tatt and Tan 1974). Fibrosis and sclerosis of the lymphatics are suggested to lead to serious regional oxygen deficiency in lymphedematous tissue, followed by periods of reperfusion (Lahdenranta et al. 2009; Lee and Kim 2024). In previous studies, decreases in GSH and increases in non-reduced glutathione (GSSG) and MDA in patients with lymphedema further suggested the accelerated production of oxygen-free radicals and increased lipid peroxidation in chronic lymphedematous tissue (Lee et al. 2022; Maruccia et al. 2019; Mizuno et al. 1998). In the present study, a comparison of tissue MDA and GSH levels showed a significant difference in Groups 2 and 3 versus Group 1, consistent with the results of previous studies. Tamoxifen is widely used in the treatment of breast cancer and has been proposed as a prophylactic agent in this disease (Nakamura et al. 2009). It has also been shown to be effective in the regression of desmoid tumors, which have mesenchymal elements similar to those of retroperitoneal fibrosis (Baik et al. 2022; Ohkawa et al. 1979; Park et al. 2013). The exact mechanism of action of tamoxifen is unknown but is thought to be related to its anti-inflammatory and anti-angiogenic properties (Baik et al. 2022). Tamoxifen is an effective antioxidant that protects membranes and low-density lipoprotein (LDL) particles against oxidative damage. It inhibits the oxidation of lipoproteins such as ceratoid, which has been implicated in the pathogenesis of retroperitoneal fibrosis (Baik et al. 2022). The antifibrotic effect of tamoxifen suggested a potential for alleviation of lymphedema, which was tested in this study, together with investigations aimed at elucidating the underlying mechanism of action. The reduced volumetric changes in Groups 2, 3, and 4, all treated with tamoxifen, compared to the untreated control group demonstrated the ability of tamoxifen to decrease lymphedema. This finding was confirmed by the fibrosis score and by the tissue hydroxyproline levels. In a previous study, tamoxifen was shown to alter the production of transforming growth factor-β, which modulates fibroblast activity and fibrosis, in soft tissues (Pritchyk et al. 2004; Rehal 2020; Rockson 2008; Sakorafas et al. 2006). Biochemical analysis of tissue samples showed that the edematous tissues of Group 2 rats contained significantly lower concentrations of MDA and significantly higher levels of GSH than those of Group 4 rats, even at 30 days postoperatively. This observation is consistent with an antioxidant effect of tamoxifen on lymphedema beginning during the early period of tissue injury. Tamoxifen is currently not prescribed for lymphedema prevention in the immediate preoperative period but may be given to patients soon after mastectomy or before they undergo radiation therapy.

The late-treatment group (Group 4) had less effective results than the early- and immediate-treatment groups. This means that more research is needed to figure out why this happened. The 14th day after surgery was probably when tamoxifen started, which was after the key early inflammatory and profibrotic phase had already begun. After a lymphatic injury, the early postoperative phase is marked by increased vascular permeability, oxidative stress, inflammatory cell recruitment, and fibroblast activation. All of these things help the extracellular matrix develop and keep the tissue remodeling going (Sakorafas et al. 2006; Scallan et al. 2016). As these processes grow more coordinated and fibrosis starts to form, the chances of therapeutic reversibility go down a lot (Sakorafas et al. 2006; Schaverien and Coroneos 2019). Their importance is shown by the fact that Group 4 had higher levels of MDA in the tissue and blood, lower levels of GSH in the tissue, bigger limbs, and more obvious fibrotic alterations than the other intervention groups. When given during the first phase of injury response, tamoxifen is more effective because it can change the way inflammatory and fibrotic signaling pathways work, leading to reduced fibrosis and improved tissue healing outcomes. It is not as effective in the second phase, when structural changes in the tissue have already happened (Scallan et al. 2016; Schaverien and Coroneos 2019) because the established fibrosis and inflammation can limit the drug’s ability to reverse or mitigate these changes.

There was no significant difference in tissue NO levels between the treated and untreated groups. NO decreases the contraction amplitude of the lymphatic pump and inhibits pump activity (Shirasawa et al. 2000; Siems et al. 1999, 2002; Suzuki et al. 2023; Tabibiazar et al. 2006; Talarico et al. 1991; Warren et al. 2007; Witte et al. 2001). In a previous study using lymphangiography and intravital microscopy to quantify lymph flow, we found that lymph flow was decreased in endothelial nitric oxide synthase (eNOS)–knockout mice (Shirasawa et al. 2000; Wu et al. 2025). Since measurements of NO levels in tissue and in blood did not show significant difference between any of the groups with surgically induced lymphedema in the present study, the effect of tamoxifen is probably not related to NO content, although NO is known to affect the function of the microlymphatic system. We also investigated the effects of tamoxifen on TNF-α levels in the four groups. Overall tissue TNF-α levels in the tamoxifen-treated groups did not differ significantly from those of the control group. However, subgroup analysis showed that serum TNF-α levels were significantly lower in Groups 2 and 3 than in Group 4. In previous studies, molecular characterization of whole-tissue homogenates from mice with lymphedema revealed high levels of inflammatory mediators such as TNF-α (Schaverien and Coroneos 2019; Wynn 2008; Yang et al. 2021; Zampell et al. 2012). These results suggest an amelioration of inflammation in the peri-operatively treated animals, although without the sham operation group, it is not possible to definitively ascribe this effect to tamoxifen treatment.

Based on our results, tamoxifen treatment was associated with a smaller postoperative limb volume increase and reduced histopathologic fibrosis, supported by hydroxyproline measurements. The lower MDA and higher GSH levels in tamoxifen-treated groups are consistent with reduced lipid peroxidation and improved antioxidant capacity, which may contribute to decreased fibrotic remodeling. Tamoxifen is traditionally administered to patients after chemotherapy and radiotherapy. While this protocol may be slow to change, our results strongly suggest that tamoxifen therapy may be beneficial when started in the immediate postoperative period, but more evidence should be produced to validate these findings before clinical studies could be justified. Furthermore, our study had methodological flaws, notably the absence of a sham surgery group, despite the presence of a control group that did not receive tamoxifen. Our study did not incorporate a sham-operated group (skin incision and tissue handling without lymphadenectomy), which would have facilitated the differentiation between nonspecific postoperative edema and lymphatic insufficiency-related swelling. In addition, measurements were obtained at baseline and day 30 but serial assessments would have better characterized the temporal course of swelling and tissue remodeling. Finally, lymphoscintigraphy did not reveal significant differences, which limits conclusions regarding lymphatic transport. Thus, the observed benefits likely reflect antifibrotic/antioxidant effects on postoperative tissue changes after tamoxifen treatment. Tamoxifen attenuated postsurgical limb swelling and soft-tissue fibrosis in this rat lymphatic injury model, accompanied by reduced oxidative stress markers. Of note, definitive prevention of lymphedema cannot be concluded from the current assessments. Our findings remain to be confirmed in further experimental and later clinical studies.

## Data Availability

All source data for this work (or generated in this study) are available upon reasonable request.
